# DNA-aided identification of *Culex* mosquitoes (Diptera: Culicidae) reveals unexpected diversity in underground cavities in Austria

**DOI:** 10.1007/s00436-019-06277-y

**Published:** 2019-03-27

**Authors:** Carina Zittra, Otto Moog, Erhard Christian, Hans-Peter Fuehrer

**Affiliations:** 1 Institute of Parasitology, Department of Pathobiology, University of Veterinary Medicine Vienna, Veterinaerplatz 1, 1210 Vienna, Austria; 2 Institute of Hydrobiology and Aquatic Ecosystem Management, University of Natural Resources and Life Sciences, Gregor-Mendel-Straße 33/DG, 1180 Vienna, Austria; 3 Institute of Zoology, University of Natural Resources and Life Sciences, Gregor-Mendel-Straße 33, 1180 Vienna, Austria

**Keywords:** *Culex pipiens* s. L., Hybrids, *Culex torrentium*, Caves, Parietal fauna, Hibernation

## Abstract

Subterranean cavities serve as resting places and hibernation shelters for mosquitoes. In Europe, members of the genus *Culex* are often the most abundant insects on cave walls. *Culex pipiens* L., the common house mosquito, exists in two physically very similar, yet genetically and ecologically distinct biotypes (or forms, ‘f.’), namely *Cx. pipiens* f. *pipiens* and *Cx*. *pipiens* f. *molestus*. Autogeny and stenogamy of the latter form have been interpreted as adaptations to underground habitats. The epigean occurrence of the two biotypes and their hybrids was recently examined in Eastern Austria, but the hypogean distribution of the *Cx. pipiens* complex and morphologically similar non-members such as *Cx. torrentium* is unknown. Considering the key role of *Culex* mosquitoes in the epidemiology of certain zoonotic pathogens, the general paucity of data on species composition and relative abundance in subterranean shelters appears unfortunate.

For a first pertinent investigation in Austria, we collected mosquitoes in four eastern federal states. Based on analyses of the ACE2 gene and the CQ11 microsatellite locus, 150 female and three male mosquitoes of the genus *Culex*, two females of the genus *Culiseta* and a single female of the genus *Anopheles* were determined to species level or below. In our catches, *Cx. pipiens* f. *pipiens* exceeded the apparent abundance of the purportedly cave-adapted *Cx*. *pipiens* f. *molestus* many times over. Records of *Cx. hortensis* and *Cx. territans*, two species rarely collected in Austria, lead us to infer that underground habitats host a higher diversity of culicine mosquitoes than previously thought.

## Introduction

Underground spaces such as natural caves, mining galleries, tunnels and culverts (henceforth referred to as ‘caves’) are resting and hibernation shelters for numerous families of insects, including hematophagous Diptera such as Psychodidae, Ceratopogonidae and Culicidae (Obame-Nkoghe et al. 2017a; Carvalho et al. 2016).

Mosquitoes (Culicidae) include vectors of human and veterinary pathogens such as arboviruses, haemosporidians and filarioid nematodes (Norris 2004; Schoener et al. 2017; Übleis et al. 2018). Distribution and transmission of these pathogens are regulated through communities of potential vector organisms (Zittra et al. 2017a). About 10% of the 3500 known mosquito species play a major role in pathogen transmission (Becker et al. 2010; Diniz et al. 2017).

Members of the *Culex pipiens* complex are critical for the epidemiology of certain viruses that menace public and veterinary health (Brugman et al. 2018). At least, Sindbis and Sindbis-like viruses (Togaviridae), Lednice virus (Bunyaviridae) and Usutu and West Nile virus (Flaviviridae) are primarily transmitted by *Culex* species in Europe (Becker et al. 2012; Roiz et al. 2012; Fros et al. 2015). Furthermore, persistence of the West Nile virus lineage 2 throughout the winter season in Europe, facilitated by vertical transmission, is strongly linked to this species complex (Rudolf et al. 2017). Heed must be paid to the fact that different host preferences of the two *Cx. pipiens* forms and the hybrids entail distinct vector competences (Lundström et al. 1990; Vogels et al. 2016). In addition, the two forms exhibit ecological differentiation: *Culex pipiens* f. *pipiens* is basically ornithophilic (bird-biting), anautogenous (the female requires a blood meal for egg development) and eurygamous (mates while swarming in a large breeding area), whereas *Cx. pipiens* f. *molestus* is mammalophilic (preferring mammals), autogenous (can lay a first batch of eggs without a blood meal) and stenogamous (mates in restricted space without nuptial flight). Immature stages of both forms are found at epigean sites, whereas in hypogean sites only f. *molestus* has been recorded so far (Byrne and Nichols 1999). Furthermore, the two biotypes are known to hybridise in areas where they coexist (Zittra et al. 2016), which may result in populations that act as bridge vectors due to their feeding preferences (Hamer et al. 2008; Černý et al. 2011). Previous studies, not only with reference to Austria, mainly focused on epigean urban and wetland habitats, completely neglecting less accessible sites such as natural caves (Lebl et al. 2014; Zittra et al. 2016).

The mosquito species inventory of Austria currently holds 49 species of eight genera (Zittra et al. 2017b). Only a restricted number of these has been reported from both natural and man-made underground cavities in Austria, namely *Cx*. *pipiens* s.l. (recorded in several federal states: Salzburg, Upper Austria, Lower Austria, Vienna, Burgenland, Styria and Carinthia), *Cx*. *hortensis* and *Ochlerotatus geniculatus* (Carinthia), *Culiseta annulata* (Lower Austria and Burgenland) (Strouhal and Vornatscher 1975) and *Uranotaenia unguiculata* (Lower Austria) (Rudolf et al. 2015). The *Cx*. *pipiens* complex, usually referred to as the common house mosquito, consists of several taxa (Bahnck and Fonseca 2006; Farajollahi et al. 2011). In Austria, only one species of this complex has been confirmed so far: *Cx*. *pipiens* L., with the biotypes *Cx*. *pipiens* f. *pipiens*, *Cx*. *pipiens* f. *molestus* and hybrids of the two (Zittra et al. 2016). A number of *Culex* species are genetically separated from the *Cx. pipiens* complex (Farajollahi et al. 2011), yet morphologically hardly distinguishable in the female sex. In Austria, this pertains to *Cx. torrentium* (Zittra et al. 2016).

Mosquitoes differ in their hibernation strategy (Andreadis et al. 2010). While many species hibernate in immature stages, *Culex pipiens* s.l. is among the mosquitoes that enter hibernacula for resting as non-blood fed, nulliparous and inseminated females. Hibernating parous females seem to experience high mortality (Andreadis et al. 2010).

Since specimens of the genus *Culex* from underground cavities have seldom been reliably determined to species level or below, the relevance of these shelters with regard to population dynamics and a potential public health risk is hard to assess. So it seems advisable to establish culicid taxa composition and relative abundance in a previously neglected habitat type: the subterranean realm. In this study, we searched into the composition of mosquito assemblages in Austrian caves for the first time, using—in case of genus *Culex*—molecular tools. We examined whether the presence of *Cx. pipiens* is in fact much higher than the presence of any other culicid species, as the catalogue of Austrian cave animals (Strouhal and Vornatscher 1975) suggests, and whether alleged underground-adapted *Cx. pipiens* f. *molestus* are actually more abundant than *Cx. pipiens* f. *pipiens* or hybrids of the two biotypes.

## Material and methods

### Mosquito sampling

From 2015 to 2018, we collected mosquitoes in 44 caves in eastern Austria. Table 1 gives the basis data of the sampling sites. The sites were selected such as to cover the different types of cave-like habitats scattered over the federal states of Vienna, Lower Austria, Burgenland and Styria, with a geographic focus on the Vienna region. Each locality was sampled one to three times, only cave Schelmenloch was sampled monthly from January 2017 to May 2018. The effort always equated to one person-hour. We used an aspirator for collecting mosquitoes resting on the cave walls. A moistened duster proved efficient for the catch of flying mosquitoes. Small caves were completely screened for mosquitoes, bigger caves from the entrance to well beyond the innermost mosquito sampling spot (in the aphotic zone, if present). The specimens were transported in cooled plastic tubes to the lab, subsequently deep-frozen and kept in the freezer until examination. Mosquitoes were identified morphologically to species or species complex level using the identification key after Becker et al. (2010). Mosquitoes classified as belonging to the *Cx. pipiens* complex or *Cx*. *torrentium* were identified subsequently to species level or form level by means of molecular tools.

**Table 1:** Name, position and characteristics of the sampling sites

Sampling site	Cave #	Fed. prov.	Municipality	Length, aphotic (X)	Alt.	Lat.	Long.	Vertebrates present	Visitor pressure
Adlersteighöhle	1915/1	LA	Maria Enzersdorf	16	255	48.084	16.276		H
Allander Tropfsteinhöhle	1911/2	LA	Alland	177 (X)	403	48.053	16.077	A	S
Altenburgerhöhle	2921/23	LA	Bad Deutsch-Altenburg	58 (X)	154	48.140	16.908		
Antonsgrotte	1912/40	LA	Baden	54 (X)	298	48.011	16.229		
Camaldulensergrotte am Kahlenberg	Artificial	VI	Wien 19	9	396	48.273	16.331		
Durchlass B 1939 Schreiberbach	Artificial	VI	Wien 19	27	427	48.274	16.321		
Durchlass Krapfenwaldbach	Artificial	VI	Wien 19	20	329	48.266	16.332		
Einödhöhle	1914/6	LA	Pfaffstätten	87	375	48.024	16.236	A	H
Einsiedlerhöhle	1912/4	LA	Baden	14	320	48.013	16.205		
Excentriqueshöhle	1915/32	LA	Kaltenleutgeben	18	348	48.122	16.222		
Gainfarnerhöhle	1911/16	LA	Bad Vöslau	25	330	47.965	16.191		
Grafenlucke III	2911/72	BL	Winden am See	6	170	47.968	16.754		H
Grotte Gspöttgraben	Artificial	VI	Wien 19	3	367	48.259	16.313		
Grufthöhle	1912/9	LA	Baden	72 (X)	276	48.010	16.229		
Guglzipfhöhle	1869/1	LA	Berndorf	16	350	47.942	16.110		
Güntherhöhle	2921/2	LA	Hundsheim	206 (X)	265	48.121	16.936		
Höllturmhöhle	1869/7	LA	Wöllersdorf	180 (X)	355	47.867	16.174		H
Johannesbachklamm-Höhle	1861/7	LA	Würflach	30	440	47.780	16.040		
Johannstein-Halbhöhle	1915/24	LA	Hinterbrühl	2	410	48.085	16.184		
Keller am Nussberg	Artificial	VI	Wien 19	4	235	48.263	16.357		
Kohlröserlhöhle	1863/59	LA	Hohe Wand	14	820	47.840	16.065	A	H
Kolloweinhöhle	2911/37	BL	Müllendorf	10	245	47.846	16.459		
Ludlloch	2911/1	BL	Winden am See	93	190	47.970	16.754		H
Luftschutzloch	1869/35	LA	Pottenstein	7	335	47.966	16.085		
Lurgrotte, Peggau	2836/1	ST	Peggau	5975 (X)	412	47.216	15.343	A	S
Lurgrotte, Semriach	2836/1	ST	Semriach	5975 (X)	636	47.227	15.379		S
Merkensteinhöhle	1911/32	LA	Bad Vöslau	72 (X)	446	47.982	16.133		
Merkurhöhle	1917/5	LA	Kaltenleutgeben	11	326	48.123	16.217		
Mittlerer Stollen, Peggauer Wand	Artificial	ST	Peggau	2	440	47.209	15.347		
Mühlriegelhöhle	2911/46	BL	Purbach	12	174	47.920	16.675		
Muldenloch	1912/31	LA	Baden	14	325	48.013	16.228		
Müllendorferhöhle	2911/30	BL	Großhöflein	10	340	47.846	16.476		
Nördlicher Stollen, Peggauer Wand	Artificial	ST	Peggau	2 (X)	440	47.209	15.347	A	
Parabluieberghöhle	1915/25	LA	Perchtoldsdorf	6	546	48.120	16.230		
Räuberhöhle	1861/12	LA	Neunkirchen	30	393	47.752	16.068		
Römersteinbruch	Artificial	ST	Wagna	2000 (X)	305	46.749	15.549	A, B	
Schelmenloch	1911/41	LA	Bad Vöslau	60	330	47.981	16.197	A, B, C, RD	
Schüttkastenhöhle	1913/14	LA	Heiligenkreuz	17	314	48.054	16.129		
Staffelhöhle	1912/30	LA	Baden	17	325	48.016	16.225		
Stollen bei der Lourdesgrotte	Artificial	LA	Heiligenkreuz	17	314	48.054	16.129		H
Türkenbrunnen	Artificial	LA	Bad Vöslau	62	530	47.985	16.124		
Unteres Türkenloch	1869/83	LA	Berndorf	5	340	47.942	16.111		
Wasserglurn	1913/12	LA	Heiligenkreuz	57 (X)	285	48.030	16.137		
Zisterne Maurer Wald	Artificial	VI	Wien 13	3	350	48.274	16.237		

Cave # = number in the Austrian cave cadastre; Fed. prov. = federal province (*BL*, Burgenland; *LA*, lower Austria; *ST*, Styria, *VI*, Vienna) Length = total passage length (m), (X) = aphotic parts present; Alt. = altitude (m above sea level); Lat. = Latitude; Long. = Longitude; Vertebrates recorded at sampling date(s): A = *Rhinolophus hipposideros*, B = *Rhinolophus ferrumequinum*, C = *Myotis myotis*, RD = *Rana dalmatina*; Visitor pressure: S = show cave (open for guided tours), H = attraction on a hiking trail (unattended visits are common)

### DNA extraction and molecular identification

DNA was extracted from single individuals using the DNeasy Blood and Tissue Kit (Qiagen, Hilden, Germany) according to the manufacturer’s protocol. In a first step, *Cx. pipiens* forms were distinguished from *Cx. torrentium* by partial amplification of the ACE2 gene (Smith and Fonseca 2004) using primers ACEpip, ACEpall, ACEtorr and B1246s in standard PCR protocols (Zittra et al. 2016). PCR products were separated using gel electrophoresis targeting 634 bp (*Cx. pipiens* forms) and 512 bp (*Cx. torrentium*) DNA fragments. Mosquitoes, determined as members of *Cx. pipiens* complex, were subsequently identified to biotype level in a second step (Bahnck and Fonseca 2006) using primers CQ11F2, pip CQ11R and mol CQ11R in standard PCR protocols. PCR products were visualised using gel electrophoresis targeting 185 bp (*Cx*. *pipiens* f. *pipiens*) and 241 bp (*Cx*. *pipiens* f. *molestus*) DNA fragments (Bahnck and Fonseca 2006). Fragments (ca. 700 bp) of mitochondrial cytochrome c oxidase subunit I (COI) of the negative samples were partially amplified using primers H15CuliCOIFw and H15CuliCOIRv (Zittra et al. 2016) in standard protocols, to test for members of similar *Culex* species that had possibly passed unnoticed through the initial morphology-based sorting. PCR products were subsequently purified and directly sequenced by a commercial company (LGC Genomics, Germany).

## Results

### Mosquito identification

We analysed 150 female and three male mosquitoes of the genus *Culex*. The male mosquitoes belonged to *Cx. torrentium* and were all collected in June and July. Among females, *Cx. torrentium* (*n* = 69) was most abundant, followed by *Cx*. *pipiens* f. *pipiens* (*n* = 44), *Culex hortensis* (*n* = 20) and the hybrids *Cx*. *pipiens* f. *pipiens* × f. *molestus* (*n* = 13). *Culex pipiens* f. *molestus* (*n* = 3) was represented in low apparent abundance. *Culex territans* (*n* = 3) has been found rarely in natural and artificial subterranean habitats, the likewise rare species *Cx. modestus* (*n* = 1) in a single natural cave. In this study, four species, namely *Cx*. *hortensis, Cx*. *territans*, Cx. *torrentium* and a single female of *Anopheles maculipennis* s.l. were recorded for the first time in subterranean habitats in Austria (Table 2). Females of *Culiseta annulata* (*n* = 2) were sampled in a natural and an artificial site in Lower Austria.

**Table 2:** Mosquitoes (number of individuals) sampled between 2015 and 2018 per month

Month	*An. mac.* s.l.	*Cs. ann.*	*Cx. hor.*	*Cx. mod.*	*Cx. ter.*	*Cx. tor.*	*Cx. pip.* f. *mol*.	*Cx. pip*. f. *pip.*	Hybrid	Excluded	Total
Jan			7			6				1	14
Feb			4			5		3		7	19
Mar						2		1	3	1	7
Apr			1		1			1	2	7	12
May										1	1
Jun						2					2
Jul	1					2				1	4
Aug											0
Sept			1		1	6		2		4	14
Oct			3			26	2	26	7	26	90
Nov						17	1	11	1	22	52
Dec		2	4	1	1	3				7	18
Total	1	2	20	1	3	69	3	44	13	77	233

*An*. *mac*. s.l. = *Anopheles maculipennis* sensu lato, *Cs*. *ann*. = *Culiseta annulata*, *Cx*. *hor*. = *Culex hortensis*, *Cx*. *mod*. = *Culex modestus*, *Cx*. *ter*. = *Culex territans*, *Cx*. *tor*. = *Cx*. *torrentium*, *Cx*. *pip*. f. *pip*. = *Culex pipiens* form *pipiens*, *Cx*. *pip*. f. *mol*. = *Culex pipiens* form *molestus*

### Mosquito distribution and phenology

*Culex torrentium* and *Cx*. *pipiens* f. *pipiens* were found in all federal states in artificial and natural subterranean habitats, single individuals of *Cx. pipiens* f. *molestus* in Burgenland and Lower Austria at natural sites, hybrids of the two forms in Burgenland, Lower Austria and Styria, mainly in natural caves. *Culex hortensis* was found in Lower Austria and Vienna, a single *Cx. territans* in Lower Austria. Male *Cx. torrentium* were found in a natural cave as well as in a tunnel in Lower Austria.

Mosquitoes were abundant in the twilight zone near the entrance and decreased towards the inner reaches. Maximum abundance was determined in autumn. The number of individuals increased in September and decreased after a peak in October (Table 3). Mosquitoes were absent at the study sites in August.

**Table 3:** Mosquitoes (number of individuals) sampled between 2015 and 2018 per federal state. Abbreviations as in Table 2

Federal state	*An. mac. s.l.*	*Cs. ann.*	*Cx. hor.*	*Cx. mod.*	*Cx. ter.*	*Cx. tor.*	*Cx. pip.* f. *mol*.	*Cx. pip*. f. *pip*.	Excl.	Hybrid	Total
Burgenland						2	1	9	26	7	45
Lower Austria	1	2	19	1	3	46	2	26	41	5	146
Styria						2		7	1	1	11
Vienna			1			19		2	9		31
Total		2	20	1	3	69	3	44	77	13	233

## Discussion

Previous observations in Lower Austrian caves (Fritsch 1992; Wurzenberger 1996) indicate that mated female mosquitoes enter caves for hibernation from late summer on. After abundance peaks in September or October, the number of individuals drops during winter due to mortality. This conforms to our results. We found females from September to May with highest abundance in September and October. The males were collected in June and July. This proves that caves can serve as resting places also for male mosquitoes, although at another season.

The composition of the *Cx. pipiens* complex in subterranean habitats reflects the observed species composition in Austrian overground habitats: *Cx*. *pipiens* f. *pipiens* is most abundant, followed by hybrids and *Cx*. *pipiens* f. *molestus* (Zittra et al. 2016). Since *Cx*. *pipiens* f. *molestus* is described to be adapted to subterranean life (Byrne and Nichols 1999), we had expected a higher proportion of this form. Our results suggest, however, that subterranean habitats do not host significant numbers of the biotype *molestus*. It seems that the two forms are equally inclined to spend winter dormancy in caves. Further investigations in extended caves with a zone of perfect darkness, the aphotic zone and steady climate should put this statement to the proof. At the same time, the distribution of the two biotypes along the light and climate gradients could be determined.

The high abundance and frequent occurrence of *Cx*. *torrentium* is new for Austria, but the species has been reported as a winter guest in underground habitats of other European countries: Dobat (1975, 1978) and Weber (1989, 1991, 1995) published several findings in Germany; records in Norway (Kjaerandsen 1993), Sweden (Jaenson 1987) and Slovakia (Moravčík 1976) were compiled by Dvořák (2014). However, these determinations are questionable, since females of *Cx*. *torrentium* are morphologically indistinguishable from females of the *Cx. pipiens* complex. In our investigation, *Cx. torrentium*, an often neglected, mainly ornithophilic mosquito species, had the highest apparent abundance in natural as well as in artificial cavities.

Combining our results with literature, seven of the 49 culicid species currently known from Austria have been recorded in subterranean habitats so far: *Anopheles maculipennis* s.l, *Cx. torrentium*, *Cx. pipiens* (with the biotypes *Cx. pipiens* f. *pipiens* and *Cx. pipiens* f. *molestus*), *Cx. hortensis*, *Cx. territans, Culiseta annulata* and *Ochlerotatus geniculatus* (Zittra et al. 2017b; Strouhal and Vornatscher 1975). *Uranotaenia unguiculata*, a potential vector of the West Nile virus (Rudolf et al. 2015), was not retrieved in this study, although the species is present in Austria and able to hibernate in caves.

Mosquitoes usually start entering hibernacula in early autumn, but our results demonstrate that natural and man-made subterranean shelters are also used as resting places during most of the year. The source of the supposedly underground-adapted biotype *molestus* remains to be clarified. Our data in combination with previously published results (Zittra et al. 2017a) indicate that caves are neither overly significant for its occurrence, nor serve as hotspots of hybridization. Therefore, we propose that this biotype naturally occurs in low abundance in Eastern Austria. However, population genetics should be conducted to assess whether *pipiens* × *molestus* hybrids contribute to overall population dynamics of the *pipiens* complex, and to elucidate the population structure within the *pipiens* complex.

Three mosquito species rated as rare, viz. *Cx*. *torrentium*, *Cx*. *hortensis* and *Cx. territans*, were collected during this study. Individual numbers of the latter two species are too low to detect any correlation with overground abundance. The three species are generally hard to come by. A common problem in mosquito ecology is under-estimation of certain species when carbon dioxide traps are used (Beck et al. 2003; Zittra et al. 2017a). It seems that mainly non-mammalophilic species are underrepresented in surveillance studies due to the low attractiveness of these standard traps (Zittra et al. 2017a). The high numbers of *Cx*. *torrentium* in the caves are therefore quite unusual as this species is typically collected in much lower proportions (Zittra et al. 2016). We assume that these relative high abundances in caves might be due to a species-specific preference for resting and hibernating in subterranean shelters. Indeed, several species of the genera *Culex*, *Culiseta*, *Anopheles* and *Uranotaenia* are known to use caves as places for hibernation or as resting sites (Trájer et al. 2018). On the other hand, we collected low numbers of *Cx. territans* and *Cx. hortensis*, both likely non-mammalophilic, that are also described as using dark and cool places as daytime resting sites. Generally, little is known about these taxa as they are rarely collected (Zittra and Waringer 2015; Zittra et al. 2016, 2017b). Sophisticated trapping devices might be necessary to capture and investigate such rarely collected, yet not necessarily rare species (Camp et al. 2018). The species-rich genera *Aedes* and *Ochlerotatus* mainly hibernate in their immature stages (Becker et al. 2010) and were therefore, as expected, not recorded in this study. The absence of *Uranotaenia* species at the studied sites possibly relates to their limited distribution in Burgenland, near Lake Neusiedl (Lebl et al. 2014; Camp et al. 2018), while there are only single records in other federal states (Lebl et al. 2015; Zittra et al. 2017).

Since tourism and modern leisure behaviour make cave visits increasingly popular, pathogens transmitted by cave-associated mosquitoes have become a topic of research, especially in tropical regions (Obame-Nkoghe et al. 2017b; Wiwanitkit 2018). In Austria, 33 out of 16,000 surveyed caves run as show caves (Oedl and Spötl 2016), but many more subterranean sites are well-frequented local attractions without regular guiding service. In Central European caves, the hazard of a mosquito-borne infection is small, as culicids are using caves mainly for resting or hibernation. Reports on mosquito attacks in caves are rare. This is in contrast to subways and similar subterranean habitats in cities (Byrne and Nichols 1999) where the environment does not fully comply with cave conditions.

Our results corroborate the importance of natural and man-made underground cavities as hibernation sites for *Culex* species and demonstrate the significance of such habitats as resting places. Subterranean shelters house a more diverse mosquito assemblage than previously inferred from literature data. We found that the composition of underground assemblages of the *Culex pipiens* complex reflects the composition in overground habitats, without proportional increase of *Cx. pipiens* f. *molestus* in the caves. The high proportions of *Cx. torrentium* are surprising but can be related to the ecology of the species. Further rare species in our material additionally suggest the inclusion of caves in mosquito surveillance programs.
